# Reduction of Fuel Consumption and Exhaust Pollutant Using Intelligent Transport Systems

**DOI:** 10.1155/2014/836375

**Published:** 2014-06-17

**Authors:** Mostofa Kamal Nasir, Rafidah Md Noor, M. A. Kalam, B. M. Masum

**Affiliations:** ^1^Faculty of Computer Science and Information Technology, University of Malaya, 50603 Kuala Lumpur, Malaysia; ^2^Centre for Energy Sciences, Faculty of Engineering, University of Malaya, 50603 Kuala Lumpur, Malaysia

## Abstract

Greenhouse gas emitted by the transport sector around the world is a serious issue of concern. To minimize such emission the automobile engineers have been working relentlessly. Researchers have been trying hard to switch fossil fuel to alternative fuels and attempting to various driving strategies to make traffic flow smooth and to reduce traffic congestion and emission of greenhouse gas. Automobile emits a massive amount of pollutants such as Carbon Monoxide (CO), hydrocarbons (HC), carbon dioxide (CO_2_), particulate matter (PM), and oxides of nitrogen (NO*_x_*). Intelligent transport system (ITS) technologies can be implemented to lower pollutant emissions and reduction of fuel consumption. This paper investigates the ITS techniques and technologies for the reduction of fuel consumption and minimization of the exhaust pollutant. It highlights the environmental impact of the ITS application to provide the state-of-art green solution. A case study also advocates that ITS technology reduces fuel consumption and exhaust pollutant in the urban environment.

## 1. Introduction

Nowadays the energy saving issue is becoming more popular in ITS. Recent increases in fuel prices have a great impact on global economic changes. The drivers are worried about their fuel consumption according to their monthly budget. Excessive use of petroleum not only increases the budget but also emits more pollutants [1]. The Texas A & M Transportation Institute found that due to congestion, urban Americans have to travel 5.5 billion hours more and they are required to purchase an extra 2.9 billion gallons of fuel for a congestion cost of $121 billion while 56 billion pounds of additional Carbon Monoxide (CO) and greenhouse gas released into the atmosphere during urban congested conditions only in 2011. The world now suffers heavily from environmental pollution [2, 3]. Hence the reduction of fuel consumption can minimize the pollutant emission and preserve the environment clean and green [4]. Though significant research has been done by many researchers in the field of fuel and energy for alternative fuels, the vehicle industry also made some attempts to improve vehicle modernization for fuel efficiency and economically viable environment friendly technology [5, 6].

ITS can be defined as wire and wireless communications based on information and electronics technologies integrated with transportation system and vehicles [7, 8]. It is a modern technique for the green technology that not only makes a single vehicle green but also makes whole groups of vehicles green. ITS is already revolutionized in the field of transportation systems [9, 10]. ITS covers a wide variety of techniques and technologies such as real-time traffic information systems (TIS), electronic toll collection system (ETCS), and automated traffic light control system (ATLCS). It is likely to emerge as the major tool to solve surface transportation challenges over the next several decades, as an infrastructure gets built alongside physical transportation infrastructure. This system deploys communications, control, electronics, and computer technologies to improve the performance of road transportation systems [11]. ITS technologies are not visionary or futuristic; they are real, already exist in several countries today, and are available to all countries that focus on developing and deploying them. ITS is a promising technology that can be used for reducing fuel consumption and exhaust pollutant which in terms protect the environment [12]. The technologies alleviate congestion, provide advanced safety, and enhance productivity [13]. ITS application is used to minimize average distance, travel time, and traffic density estimation [14]. It can be used for green purposes by informing the driver of the best path that can reduce the significant amount of fuel as the vehicle choice is the less congested route [15].

Vehicles can send and receive message with important data and send for best path according to their location, speed, and direction [16]. An intelligent vehicle collects data using some special sensors. After processing this data, it broadcasts the information to other vehicles. Majority of vehicles in present days run on fossil fuels [17, 18]. Hence, significant improvement is necessary for ITS to reduce fuel consumption as well as pollutants which in terms prevents the global warming and greenhouse gas [19–21]. The ITS technologies promote the reduction of fuel consumption with two aspects, that is, first to reduce congestion that maintains each vehicle to optimal speeds and secondly to give a suggestion to the driver for a green fuel efficient path [22].

This paper survey is to find out the effect of ITS techniques and technologies on energy saving and reduction of environmental pollution from vehicles and road transportation systems including V2V and V2I, a green navigation system which helps to find out the best path for the minimization of fuel consumption and exhaust pollutant to provide the-state-of-art green solution, and finally a case study advocates the issues.

## 2. Literature Review

### 2.1. ITS Technology

There are a number of techniques and technologies used for the reduction of fuel consumption to make the environment greener. ITS could be used for reduction of fuel consumption which would make the environment clean and green [15].  Table 1 shows many techniques and technologies used for the reduction of fuel consumption in the road transportation system. Fuel consumption can be reduced by two ways, that is, reduction of fuel use and minimization of the average distance. Secondly, the technique on fuel consumption reduction introduces the importance of reduction of fuel consumption for green driving and reduction of fuel by intelligent driving, while minimization of the average distance can be done through traffic reduction by navigation and traffic reduction by transportation reduction. The ITS techniques and technologies can facilitate the reduction of fuel consumption by improving the driving behavior and minimizing the traffic congestion [35].

**Figure 1:**
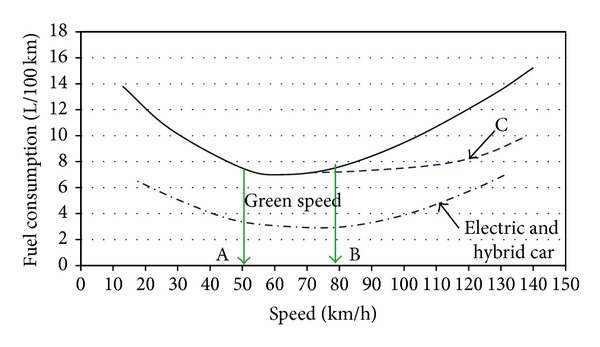
Relation between fuel consumption and average speed.

The ITS techniques and technologies can reduce energy consumption by changing the driving behavior, suggesting congestion free smooth path, automatic traffic control signal, electronic toll collection, and platooning. From the mechanical properties of the vehicle the automobile engineer proved that the vehicle running 50–70 km/h for gasoline engines and 50–80 km/h for the petrol engine consumed the lowest rate of fuel.  Figure 1 illustrates the basic relationship of the vehicle speeds with the fuel consumption from which exhaust pollutant by the driving pattern can be assumed [36, 37]. By eliminating the congestion and suggesting an uninterrupted path with the aid of ITS technique the vehicle can maintain this green speed and then obtain the best fuel efficiency and pollution at minimum level [38]. If the vehicle drives above green speed or runs bellow the green speed it will consume more fuel [39]. The curve C in Figure 1 shows that if the aerodynamic drag is reduced at high speed, then fuel consumption will also be reduced [40]. The speed versus fuel consumption for the hybrid and electric vehicle is shown by doted das line.

**Figure 2:**
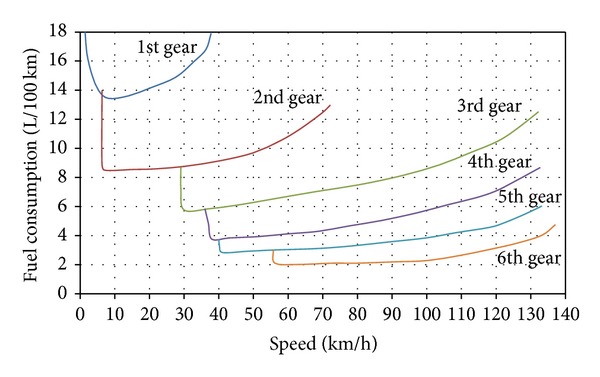
Relation between fuel consumption and gear change of a manual driving car.

Figure 2 shows how the fuel consumption varies according to gear change of a manual driving car. The best way to maintain the engine in low speed and high torque mode is to select the highest speed ratio. Engine consumes less fuel in 3rd gear than in 1st gear and less fuel in 5th gear than in 4th gear. The lower speed ratios are the most fuel guzzling because they are associated with an engine that is not sufficiently loaded. The manual transmission vehicle goes to the highest speed ratio as soon as possible. When going up a slope, avoid shifting to a lower gear as much as possible to keep engine loaded. As this approaches a stop, shift to a lower gear without braking so as to recover energy over a greater distance. With an automatic transmission, it is more difficult to control speed ratios but this can be done by momentarily taking foot off the gas pedal when going up a slope to reach the upper speed ratio.

**Figure 3:**
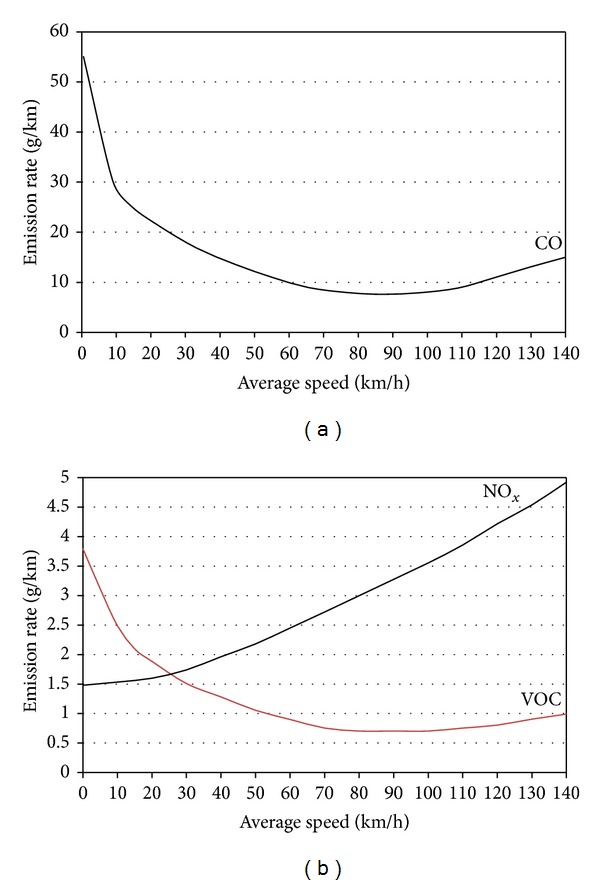
Typical relation between emission and average speed. (a) CO versus average speed and (b) NO_*x*_ and VOC versus average speed.

If automatic transmission vehicle has an optional speed ratio, activate it to obtain a higher ratio, which will reduce speed and fuel consumption. On a road with many ground level differences avoid using the speed regulator to maintain a constant speed, as the gearbox will shift to a lower speed and will increase the engine speed when going up a slope in order to maintain the same speed [41].  Figure 3 presents the vehicle emission as function of average speed [42].  Figure 3(a) shows that, at low speed, car emits the highest CO while higher speed emits minimum pollutant. The greener speed range is 60–100 km/h in terms of emission. At green speed, it emits the lowest level of CO [43].  Figure 3(b) shows the emissions of VOCs or HCs and NO_*x*_ versus average speed. Masum et al. [44] reported that NO_*x*_ increases with engine speed as more fuel is burnt resulting in high in-cylinder temperature at high speeds. NO_*x*_ emission increases more than linearly with the increase of average speed [45, 46]. At lower speed NO_*x*_ emission is lower but HC and CO emissions are higher. Rich fuel-air mixture and incomplete combustion are the reasons behind higher CO and HC emission at lower engine speed. Few authors [47, 48] get higher CO and HC emission at lower engine speed. At higher engine speed, CO and HC emissions are also higher [37]. At higher engine speed, the air-fuel mixture gets a shorter time to complete combustion that results in higher HC and CO emissions [44]. Finally we can conclude by analyzing all those graphs that 60–80 km/h is the best average speed both in terms of energy efficiency and in terms of greener environment.

### 2.2. Fuel Saving ITS Application

A number of ITS applications have to reduce the fuel consumption and exhaust pollutant. The ITS related technologies are described below.

#### 2.2.1. Intelligent Traffic Signal Control

**Figure 4:**
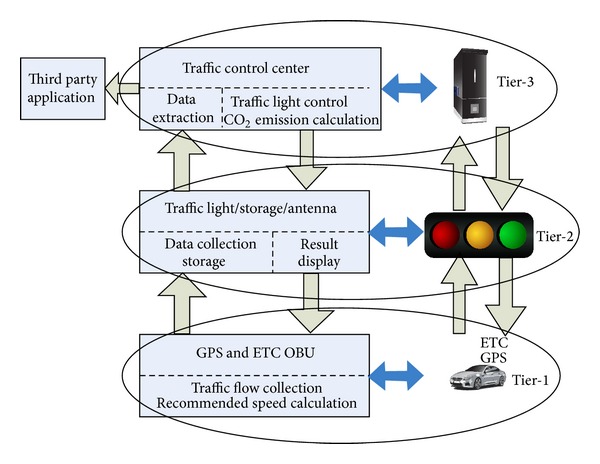
Three-tier open traffic control system.

The ITSC system plays an essential role in both safety and efficiency of road traffic [30]. The target of the ITSC system is the reduction of congestion queue time in traffic signal. ITSC reduces the waiting time in traffic control signal [49]. ITSC uses a wireless communication between RSU and vehicle [50]. The effects of ITSC are the reduction in congestion, the economic effect, and the reduction of pollutant. Vehicles in a stop-and-go running consume more fuel and emit more pollutants than constant speed driving. Very low average speeds generally represent stop-and-go driving and vehicles do not travel far. Therefore, the emission rates per mile are quite high. When a car's engine is running but it is not moving, its emission rate per mile reaches infinity [51]. Vehicles need to be smoothed for reducing CO_2_ emissions by minimizing the stop-and-go times. Wen [52] proposed a three-tier dynamic TLC system structure to minimize the emitted pollutant by uninterrupted driving. Maslekar et al. [53] proposed an ITLC system which assumed that every vehicle will be equipped with GPS, OBU, and navigation system. GPS devices collect all the information about the vehicle and road present status. OBU devices send information about the vehicle speed, acceleration, and direction by WAVE. The ETC center processes all the information and reasoning by ITLC algorithm. The brief description of three-tier open traffic light control model [54] is shown in Figure 4.

- Tier-1: tier-1 is responsible for collecting traffic information, receiving light phase data, and sending traffic flow data and it also calculates the suggested speeds. GPS devices will provide the vehicle state information. To transmit the current traffic information to ITSC, vehicle uses the OBU devices. The OBU will calculate the recommended speed when vehicles get the traffic information from the traffic lights. By using the ITSC the drivers may minimize the waiting time and also minimize number of stops.
- Tier-2: tier-2 controls the receiving and saving of traffic flow data and sends the control result to the ITSC from the OBUs. It has three parts, that is, antennas, storage, and traffic lights. The ETC's OBU devices antennas in tier-1 can communicate with other devices by wireless communications; hence, the traffic light will receive the real-time traffic flow information. At the same time, the traffic control results will be sent to ECT's OBU and then drivers can know the traffic light phases in time. The purpose of the storage is to save the received traffic flows data. The traffic lights are the displays that show the control results.
- Tier-3: data processing task is done in tier-3 from the three sections. Data extraction is in Section 1. The antenna periodically accepts the traffic information from the vehicles. Data processing task is done in this tier and data is fed from the tier-2 of ITSC. Road traffic flow data is collected by ETC system and recommends the best speed. An open interface for third-party application is operated at Section 3.

#### 2.2.2. Electronic Toll Collection Systems (ETCS)

**Figure 5:**
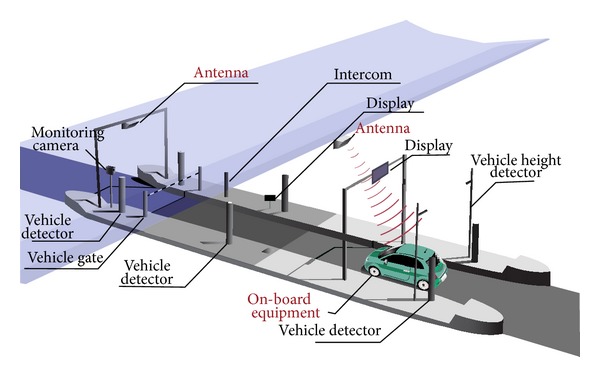
Electronic toll collection system.

ETCS is a system that permits for collection of toll payments and traffic monitoring electronically by uninterruptedly of vehicle moving [23]. ETCS have several parts for operating such as wireless communication, in-road/roadside sensors, electronic tags, and vehicle equipped with onboard equipment. ETCS provides general vehicle monitoring and data collection and collects the tolls. ETCS operate while vehicles run at near-highway cruising speed for collecting the tolls and increase efficiency and reduce congestion and travel time and reduce pollution. ETCS makes the toll gates less congested and as a result reduces the exhaust pollutant. The annual pollutant emission will be reduced to half if the urban expressway network uses ETCS.  Figure 5 shows a typical ETCS system.

By using the ETCS, the factor of CO, HC, and NO_*x*_ levels is reduced at a significant level. This analysis also showed that the air pollution emission levels at the toll booth links are reduced for all pollutants.

#### 2.2.3. Traffic Information System

TIS is very important for ITS application. The information about the number of vehicles in the road is very important to eliminate the traffic congestion. The traffic information system gathers the traffic data and transmits this data to the driver in the roads [55]. In VANET, every vehicle periodically exchanges information every 300 ms. The traffic density is the most influential factor that affects the average speed of the vehicle [56, 57]. ITS application performance depends on how accurately it can measure the traffic flow rate, traffic density, and mean speed of the vehicle. VANET is a high mobility network that greatly affects the green measures. Fuel consumption varies due to different speeds, accelerations, stop-and-go times, different followed routes, and the level of traffic congestion.

#### 2.2.4. Cooperative Driving

The cooperative driving is an automatic driving of over 2 or 3 lanes used for openly lane changing, merging, and splitting for congestion free driving. The main aim of the cooperative driving is to save the energy and to minimize the air pollution [55]. It is a vehicle-to-vehicle based communication [58]. The system was tested first in 1997 by the AETAT using the V2V infrared signal [59]. The distance between the vehicles was measured using triangulation between a pair of infrared markers on the top of a preceding vehicle during cooperative driving. In the cooperative driving application the requirement for the V2V communication is the compatibility of the real-time data transmission required for automated driving.

#### 2.2.5. Platooning

The platooning can be defined as a collection of vehicles that travel together and actively coordinate information [60]. Platooning offers a list of advantages including increase of fuel and traffic efficiency, safety, and driving comfort. The main goal of the platoon is to be relieved from the traffic congestion by vehicle automation technology. It operates each vehicle close together with compare to manual driving condition; hence every lane can carry approximately double the traffic than current manual system. This obviously shrinks the traffic congestion in highway. It maintains a close spacing aerodynamics drag that results in a major reduction in fuel consumption and exhaust pollutant. Result has shown how that drag reduction improves the fuel efficiency and emission reduction by 20 to 25%. For these reasons a number of platooning projects have been continuing such as SARTRE [61], a European platooning project; PATH [60], a California traffic automation program that includes platooning; GCDC [62], a cooperative driving initiative; SCANIA [60] platooning and; Energy ITS [63], a Japanese truck platooning project.

The summary of the ITS applications is given in Table 2.

## 3. Proposed Fuel-Saving Navigation System

Design of dynamic green driving advisor should satisfy the following goals and requirements.

- Use ITS techniques and technologies to gather the real-time traffic information and the green navigation system will update the traffic information to modify the planned path adaptively.
- Calculate accurately the vehicle flow rate based on the traffic flow theory.
- To estimate the vehicle density on specific time use historical traffic information.
- Try to maintain the average green speed (50–80 km/h) to get fuel efficiency as well as pollutant at minimum level.
- Design of dynamic speed limit should satisfy the goals and requirements of green driving.
- The strategy should work even when only one vehicle is doing green driving; more vehicles doing green driving would smooth traffic better.

### 3.1. Model Assumption

To achieve the objective behind developing a fuel efficient route selection model, some assumptions need to be agreed on to fulfill the requirements. For example, each vehicle is equipped with a set of devices, which are considered to be available on the vehicles at the present time. These include the OBU, preloaded digital road maps, GPS, and NS. Each vehicle equipped with OBU system collects its own traffic information, including location, spacing, velocity, and acceleration, from GPS device [64]. It is also able to communicate with other vehicles equipped with IVC system by DSRC. Hence, vehicles in transportation system can share their information based on this information; drivers can decide their driving behaviors to smooth traffic. An efficient fuel saving navigation system estimates the green optimum path [37]. A green navigation system provides suggestion for fuel efficient route to driver based on available information about fuel dependent parameter of each vehicle for unraveling traffic congestion. When a driver plans to go on a destination, he sends a query to navigation server with vehicle position and destination by ITS. The server will find the best fuel efficient paths to destination considering current and historical traffic data. In ITS technology, a number of sensors are installed in the road section to find out the vehicle density, traffic flow rate, and the vehicle mean speed. The next section shows the mathematical model of how to calculate those three, that is, the vehicle density, traffic flow rate, and the vehicle mean speed.

### 3.2. Vehicle Density

Vehicle density referred to the number of vehicles per kilometer in a specific time. Vehicle density *ρ* measures the number of vehicles at location *S* in certain time interval and can be measured for a road section with Δ*X* length as

(1) $$\begin{array}{c} \rho =\frac{n}{\Delta X}. \end{array}$$

The vehicle density *ρ* varies with location and time. So considering those parameters in (1) it can be written as

(2) $$\begin{array}{c} \rho \left(x_{1},t_{1},S_{1}\right)=\frac{n}{\Delta X}, \end{array}$$

where *x*_1_ is the measured location and *t*_1_ is the time interval and *S*_1_ is the road section. Normally the unit of the vehicle density is vehicles per kilometer. Now we can make a general form by multiplying numerator and denominator of (2) by a small time interval* dt*. Consider

(3) $$\begin{array}{c} \rho \left(x_{1},t_{1},S_{1}\right)=\frac{n\cdot dt}{\Delta X\cdot dt}. \end{array}$$

The numerator of (3) is the total number of vehicles in *S* at time *t* and the denominator shows the area of the measurement interval *S*. So the vehicle density for a measurement interval *S* at location *x* and at time *t* can be written as

(4) $$\begin{array}{c} \rho \left(x,t,S\right)=\frac{\text{Total}\;\text{Number}\;\text{of}\;\text{Vehicles}\;\text{in}\;S\;\text{at}\;\text{Time}\;t}{\text{Area}\left(S\right)}. \end{array}$$

### 3.3. Vehicle Flow Rate

Vehicle flow rate is the number of vehicles that pass through a certain road section per time unit. The vehicle flow rate Φ at location *x*_2_ and a time interval Δ*T* of measurement interval *S*_2_ can be defined as follows.

For a time interval Δ*T* at any location *x*_2_, the flow rate is

(5) $$\begin{array}{c} \Phi \left(x_{2},t_{2},S_{2}\right)=\frac{m}{\Delta T}. \end{array}$$

The number *m* is the total number of vehicles that pass through the location *x*_2_ during Δ*T*. The unit of vehicle flow rate is vehicle per hour. Multiplying the numerator and the denominator by a small location interval* dx* we find a more general form for vehicle flow rate. The numerator becomes the total distance travelled by all vehicles and the denominator is the area. Consider

(6) Φ(x2,t2,S2)=m·dxΔT·dx=Total  Distance  Covered  by  Vehicles  in  S2Area(S2).

From (6) we can find the general definition for vehicle flow rate as follows:

(7) Φ(x,t,S)=m·dxΔT·dx=Total  Distance  Covered  by  Vehicles  in  SArea(S);

*S* is the total distance covered by the vehicle.

**Figure 6:**
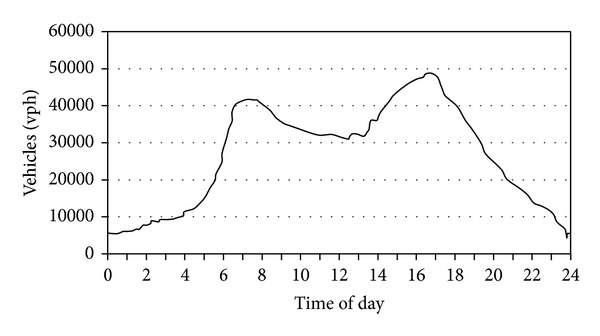
Typical traffic flow versus time of day.

The vehicle flow rate versus hour report provides a graph report that shows the historical traffic flow volumes and average speed of the transportation network during a selected time period of the day. This information is useful for analyzing the historical performance of the transportation network and implementing proactive measures to improve the flow of traffic and it is useful to make a decision for green route selection.  Figure 6 shows a typical traffic flow versus time of day.

### 3.4. Vehicle Mean Speed

The vehicle mean speed *μ* can be defined as the average speeds of all the vehicles for a location in a certain interval. The vehicle mean speed also depends on location, time, and measurement intervals. We can make a relationship with vehicle density and vehicle flow rate as follows:

(8) μ(x,t,S)=q(x,t,S)k(x,t,S)=Total  distance  covered  by  vehicles  in  STotal  time  spent  by  vehicles  in  S.

From (8) we can rewrite the vehicle mean speed as the fundamental relation of traffic flow theory as follows:

(9) $$\begin{array}{c} \Phi =\rho \cdot \mu . \end{array}$$

This is the general relation among vehicle flow rate, density, and mean speed. Using this equation, by knowing two of these variables, we can easily find the third variable. The vehicle mean speed for total *n* vehicles in the interval *S* at location *x* and point in time *t* can be calculated as

(10) $$\begin{array}{c} \mu \left(x,t,S\right)=\frac{1}{n}\sum_{n}v_{i}. \end{array}$$

From (4) and (7) we can easily find the mean speed

(11) $$\begin{array}{c} \mu \left(x,t,S\right)=\frac{1}{\left(1/m\right)\sum_{m}\left(1/v_{f}\right)}. \end{array}$$

## 4. Methodology

The proposed green fuel efficient route choice procedure uses different ITS technologies. The green navigation method finds the multiple candidates for a specific journey and chooses the most fuel efficient route. The method avoids manual traffic signal and toll collection and does not select a route to a destination in which a traffic jam might happen. The most fuel efficient route between sources to destination may be different from the shortest and fastest routes. There are several factors that affect the fuel consumption on streets. These parameters are classified into four categories, that is, static street parameters, dynamic street parameters, car specific parameters, and personal parameters. Static street parameters model the street characteristics and do not change (or change very infrequently) over a period of time. For example, the speed limits of streets change very infrequently and the number of traffic lights on the street remains more or less constant. The dynamic street parameters are characteristics that change with time. for example, the congestion levels on a street or the average speed on a street. The static and dynamic street parameters together determine the fuel efficiency of a particular street. Other variations in the fuel consumption can occur due to the type of car being driven and the nature of the person's driving. For example, a big car may consume more fuel than a small car. Similarly a person who is more erratic (higher acceleration or hard braking) is likely to consume more fuel than a more “careful” driver. These parameters account for the variation in fuel consumption due to the car type and the driver behavior. The proposed system is a linear model that can accurately predict the fuel consumption across urban traffic streets. We will summarize this model below. The input to the model includes

- static street parameters: number of stop signs (ST) from source to destination;
- dynamic street parameters: *v*, *v*_2_, and *v*_3_, where *v* is the vehicle means speed on a specific street.

### 4.1. Mathematical Model

The mean speed can be obtained from (11).

Total fuel consumption that a vehicle consume in an urban journey is fuel consume at while running and consume at stop sign. Consider

(12) Total  fuel  consumption=fuel  consume  at  running   +consume  at  stop  sign⁡.

The final model is expressed as

(13) $$\begin{array}{c} \text{Total}\;\text{fuel}\;\text{consumption}\;\text{TFC}=\overset{n}{\underset{i}{\sum }}s_{i}v_{i}+f_{c}\overset{m}{\underset{j}{\sum }}t_{j}, \end{array}$$

where TFC = Total  fuel  consumption, *S*_*i*_ = length of road section* i* (*S*_*i*+1_ − *S*_*i*_), *v*_*i*_ = mean speed of road section *S*_*i*_, *f*_*c*_ = fuel consumption per second while vehicle at idle, and *t*_*i*_ = idle time at point *j*.

### 4.2. Material and Methods

As stated before, the shortest path route or minimum travel time route may not always be the fuel efficient path. Street congestion, elevation variability, average speed, and average distance between stops (e.g., stop signs) lead to changes in the amount of fuel consumed making fuel efficient routes potentially different from the shortest or fastest routes and a function of vehicle type. To experiment and analyse the fuel saving model, a pair of source destinations with multiple routes at Kuala Lumpur was selected. Experiment was done in three different scenarios, that is, free flow condition, moderate congestion, and heavy congestion.

**Figure 7:**
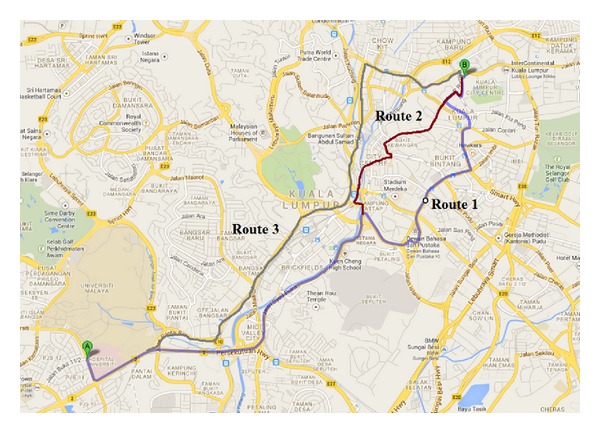
Three different routes of the same origin and destination.

Figure 7 shows three different routes from the source point A to destination point B. The distance of route 1 is 12.1 km, route 2 is 10.8 km, and route 3 is 11.2 km. From Figure 6 it was shown that 10:00 pm to 7:00 am the road in free flow condition. During 10:00 am to 2:00 pm of the day is moderate congestion where as heavy congestion occur two time slot of the day; first one is morning office time from 7:00 am to 10:00 am and second one is 4:00 pm to 9:00 pm.

## 5. Result and Discussions

### 5.1. Free Flow Condition

**Figure 8:**
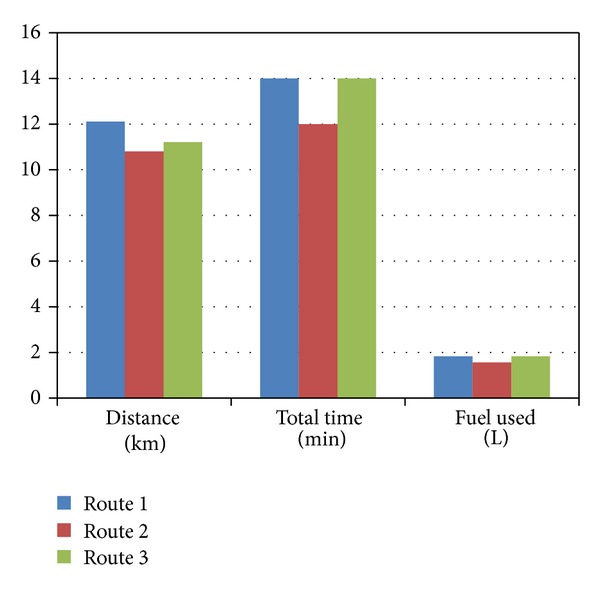
Bar graph for the distance, total travel times, and fuel used in free flow condition.

By illustrating the free flow condition, the shortest distance route 2 is also fuel efficient and also emits relatively lower pollutant.  Table 3 shows all the data found in free flow condition in three different routes.  Figure 8 shows the bar graph for the distance, total travel times, and fuel used in free flow condition in three different routes.

### 5.2. Moderate Congestion

To demonstrate the moderate congestion condition, Table 4 shows the detailed data of this case study. Normally at the noon time the congestion of the road is tolerable and the traffic density of the road is at random manner. This time route performs the most fuel efficient and environment friendly; it may differ from other times.

**Figure 9:**
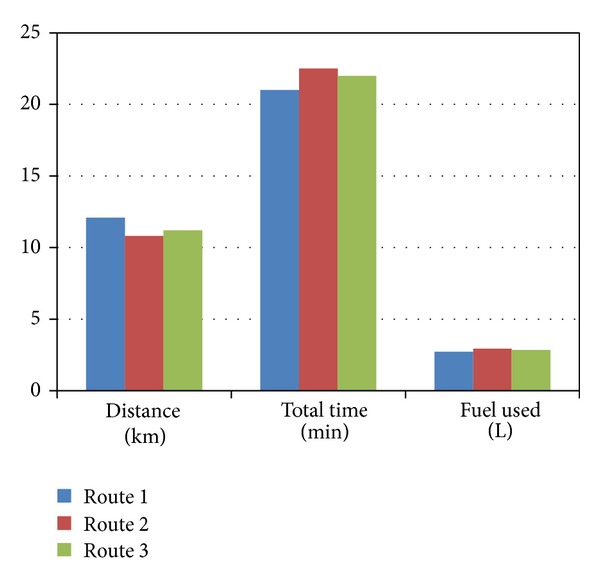
Bar graph for the distance, total travel times, and fuel used in moderate congestion.

Figure 9 shows the bar graph for the distance, total travel times, and fuel used in average congestion in three different routes.

### 5.3. Heavy Congestion

**Figure 10:**
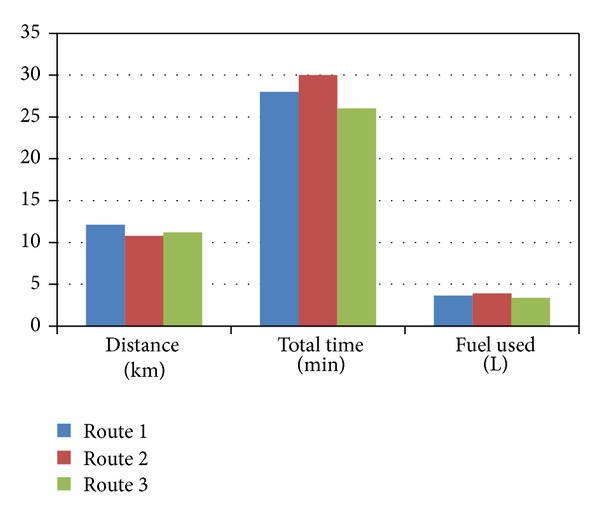
Bar graph for the distance, total travel times, and fuel used in heavy congestion.

In a heavy congested condition the road is very rushy as at morning most of the travelers go for work and at after noon they go back home from work.  Table 5 shows the details of the study; route 3 is more fuel efficient than the other two routes though route 2 is the shortest route.  Figure 10 shows the bar graph for the distance, total travel times, and fuel used in heavy congestion in three different routes.

## 6. Conclusion

Green technology is one of the most important considerations on developing ITS, foster environmental sustainability, and the economics of energy efficiency. The important issues of green technologies are related to energy efficiency in automobile industry and promote environment friendly communication technologies and systems. Green ITS technologies play a significant role in reducing energy consumption in automobile and road transport system for a variety of applications. This paper provides a survey on the effects of ITS related techniques on the reduction of fuel consumption and exhaust pollutant. In ITS, most of the applications are for highlighting traffic safety and infotainment. However, this research work sorts out ITS technologies that deploy for fuel saving and green environment. Finally, this research proposed a green navigation technology that used the current traffic flow data as well as historical traffic information. A case study shows that if the driver uses the green navigation system, it will save fuel and reduce the environment pollution. For short distance and single vehicle it shows a little impact, but if it is considered for long distance and millions of vehicle it will have significant contribution in terms of energy and environment.

## Figures and Tables

**Table 1:** Techniques and Technologies for fuel reduction of vehicle.

Reduction Parameter	Reduction Type	Attribute	Techniques		Technologies
Fuel Reduction	Importance of Reduction of Fuel Consumption for Green Driving	Vehicles	Improvement of Fuel Efficiency of Vehicle By Upgrading Mechanical Properties		Upgrading Mechanical Properties
		Roadways	Improvement of Highways		Upgrading Civil Properties
	Reduction of Fuel by Intelligent Driving	Green Driving Behavior	Maintain Optimum Tire Pressure		
			Adjust Drive Technique		
			Maintain The Ride		
			Get Rid of Weight and Reduce the Drag		
			Avoid Unnecessary Idling		
			Use Latest Technology Car		
		Traffic Flow	Intelligent Management of Highways	Lane	
				Electronic Toll Collection	
				Traffic	Traffic Light Control
				Collision Avoidance	
				Maximize Throughput	Intelligent Navigation System
			Bottleneck Elimination		Electronic Toll Collection

Shortest Distance	Traffic Reduction by Navigation	Increase Transportation Efficiency	Occupancy Increase		Car Sharing, Car Pool,
		Other Effective Factor For Transportation	Multi-Modality		Public Transportation
	Traffic Reduction by Transportation Reduction	Minimization Of Transportation	Demand Management		Road Pricing
			Parking Strategies		
		No Transportation	Communication		VANET
			City Planning		Compact City

**Table 2:** Summary of ITS application.

Authors	Application	Technology	Objectives
Fuyama [23]	Electronic toll collection System (ETCS)	Wireless communication between a roadside antenna in a tollgate and a vehicle unit in a moving vehicle	Maintain a constant green speed in toll gate
Tengler and Heft [24]	Vehicle Information Communication Systems (VICS)	Provide the traffic and travel data to the drivers by transmitting using wireless technology.	Reducing traffic congestion, traffic accidents, and improving road environment
Glass et al. [25]	Traffic Management Systems (TMS)	TMS include onboard satellite navigation devices as well as dynamic driver assistance and variable message signs.	Transport can be made safer, cheaper, more reliable and greener.
Boatright et al. [26]	Vehicle Navigation System (VNS)	Uses information from a Global Positioning System (GPS) to obtain velocity vectors, which include speed and heading components.	Advice the driver for the shortest and fuel efficient path.
Pfeiffer et al. [27]	Driver Assistance Systems	Based on intelligent sensor technology constantly monitor the vehicle surroundings as well as the driving behavior.	Detect potentially dangerous situations at an early stage and actively support the driver
Hoeger et al. [28]	Automated Driving System	Real-time driving functions necessary to drive a ground-based vehicle without real-time input from a human operator.	Traffic-jam reduction and full-range automated cruise control
Masum et al. [29]	Urban Traffic Information Systems (UTIS)	Create, analyze and process the location information of moving vehicle to improve convenience by providing improved flow of transportation logistics and analyzed traffic information to driver.	Total management system of the streetlight light and security light and reduction of pollution
Wiering et al. [30]	Intelligent Traffic Light Control System.	Intelligent traffic light control system comprising a microprocessor, a manual input device, an enforced switching device and an intelligent detecting device, where in the microprocessor is used for controlling traffic lights.	Maximize the traffic efficiency of intersection of roads and achieving a best control for traffic.
Lemelson and Pedersen [31]	Vehicle Collision Avoidance System	It uses radar and sometimes laser and camera sensors to detect an imminent crash.	To reduce the severity of an accident which in term reduce congestion.
de Fabritiiset al. [32]	Traffic Estimation and Prediction System	Use computer, communication, and control technologies to monitor, manage, and control the transportation system.	Improve traffic conditions and reduce travel delays.
Smith, et al. [33]	Scalable Urban Traffic Control	The SURTRAC dynamically optimizes the control of traffic signals in three sections: first, decision making in decentralized manner of individual intersections; second is an emphasis on real-time responsiveness to changing traffic condition and finally managing urban road networks.	Objectives include less waiting, reduced traffic congestion, shorter trips, and less pollution.
Blum et al. [34]	Intelligent Speed Adaptation (ISA)	There are four types of technology used for ISA: GPS, Radio Beacons, Optical recognition, Dead Reckoning	ISA helps to reduction of accident risks and reductions of noise and exhaust emissions.

**Table 3:** Free flow Condition Fuel Consumption.

Performance Measure	Route 1	Route 2	Route 3	Remarks
Distance (Km)	12.1	10.8	11.2	
Running time (Minutes)	12 m	11 m	12 m	
Stop time (Minutes)	2 m	2 m	2 m	
Total time (Minutes)	14 m	13 m	14 m	
Total distance w.r.t. time	14 Km	13 Km	14 Km	Assumption-1
Fuel used (Liter)	1.82	1.69	1.456	
Fuel consumption (Lt/Km)	0.13	0.13	0.13	

**Table 4:** Performance on moderate congestion road condition.

Performance Measure	Route 1	Route 2	Route 3	Remarks
Distance (Km)	12.1	10.8	11.2	
Running time (Minutes)	17 m	18 m	18 m	
Stop time (Minutes)	4 m	4.5 m	4 m	
Total time (Minutes)	21 m	22.5 m	22 m	
Total distance w.r.t. time	21 Km	22.5 Km	22 Km	Assumption-1
Fuel used (Liter)	2.73	2.925	2.86	
Fuel consumption (Lt/Km)	0.13	0.13	0.13	

**Table 5:** Performance on heavy congested road condition.

Performance Measure	Route 1	Route 2	Route 3	Remarks
Distance (Km)	12.1	10.8	11.2	
Running Time (Minutes)	20 m	21 m	18 m	
Stop Time (Minutes)	8 m	9 m	8 m	
Total time (Minutes)	28 m	30 m	26 m	
Total distance w.r.t. time	28 Km	30 Km	26 Km	Assumption-1
Fuel used (Liter)	3.64	3.9	3.38	
Fuel Consumption (Lt/Km)	0.13	0.13	0.13	

## Nomenclature

AETAT: Association of electronic technology for automobile traffic
ADS: Automated driving system
ATLCS: Automated traffic light control system
CMEM: Comprehensive modal emissions model
CO: Carbon monoxide
CO_2_: Carbon dioxide
DAS: Driver assistance systems
DSRC: Dedicated short range communication
EPA: Environmental protection agency
ETCS: Electronic toll collection system
ETC: Electronic traffic control
FTP: Federal test procedure
GHG: Greenhouse gas
GNS: Green navigation system
GPS: Global position system
HC: Hydrocarbons
ISA: Intelligent speed adaptation
IT: Information technology
ITLCS: Intelligent traffic light control system
ITS: Intelligent transport system
IVC: Intervehicle communication
LDT: Light duty truck
LSR: Least square regression
MEC: Modal emission cycle
MoE: Measures of effectiveness
NO_*x*_: Oxides of nitrogen
NS: Navigation system
OBU: On-board unit
ORNL: Oak Ridge National laboratory
RSU: Road side unit
SURTRAC: Scalable urban traffic control
TEPS: Traffic estimation and prediction system
TIS: Traffic information systems
TMS: Traffic management systems
UTIS: Urban traffic information systems
V2I: Vehicle-to-infrastructure (V2I)
V2V: Vehicle-to-vehicle
VANET: Vehicular ad hoc network
VCAS: Vehicle collision avoidance system
VICS: Vehicle information communication systems
VMT: Vehicle miles travelled
VNS: Vehicle navigation system
VOC: Volatile organic compounds
WAVE: Wireless access for vehicular environment.
